# Supplementary material for: The genome sequence of a springtail, Orchesella flavescens (C.Bourlet, 1839)
Source: Wellcome Open Res. 2025 Sep 26;10:138. Originally published 2025 Mar 18. [Version 2] doi: 10.12688/wellcomeopenres.23773.2 (PMC12476540; doi:10.12688/wellcomeopenres.23773.2)
Supplement: Peer review history

# Reviewer report: Reviewer response for version 2

**DOI:** 10.21956/wellcomeopenres.27550.r134423

**Published:** 2025-10-09

**Authors:** Nerivânia Nunes Godeiro

**Affiliations:** 1 Shanghai Natural History Museum, Shanghai, Shanghai, China

I have no further comments.

Are sufficient details of methods and materials provided to allow replication by others?

Partly

Is the rationale for creating the dataset(s) clearly described?

Partly

Are the datasets clearly presented in a useable and accessible format?

Yes

Are the protocols appropriate and is the work technically sound?

Yes

Reviewer Expertise:

Collembola phylogenomics and taxonomy

I confirm that I have read this submission and believe that I have an appropriate level of expertise to confirm that it is of an acceptable scientific standard.

# Reviewer report: Reviewer response for version 2

**DOI:** 10.21956/wellcomeopenres.27550.r134421

**Published:** 2025-09-27

**Authors:** Michael Hiller

**Affiliations:** 1 Senckenberg Nature Research Society, Frankfurt Am Main, Germany

All my comments have been addressed.

Are sufficient details of methods and materials provided to allow replication by others?

Yes

Is the rationale for creating the dataset(s) clearly described?

Yes

Are the datasets clearly presented in a useable and accessible format?

Yes

Are the protocols appropriate and is the work technically sound?

Yes

Reviewer Expertise:

genomics

I confirm that I have read this submission and believe that I have an appropriate level of expertise to confirm that it is of an acceptable scientific standard.

# Reviewer report: Reviewer response for version 1

**DOI:** 10.21956/wellcomeopenres.26221.r120913

**Published:** 2025-04-07

**Authors:** Michael Hiller

**Affiliations:** 1 Senckenberg Nature Research Society, Frankfurt Am Main, Germany

The study presents the first high-quality genome of a springtail in the Genus Orchesella. The genome is highly contiguous and complete. Also, I am pleased to see that the PacBio library preparation protocol is described in detail.

 I have only one comment regarding the scaffolding: A karyotype is likely not known (?) and the HiC signal shows variation inside the chromosome-level scaffolds. E.g. scaffold 2 and 5 have a block at the bottom that equal signal to the other regions in same same and other scaffolds. Scaffold 1 also has a smaller part at the bottom that appears different. I therefore wonder if one can check if the 6 chromosomes have telomers and if the potentially problematic regions lack telomers.

 What is meant by 'dubious records' in the introduction? Species misidentification?

 It would be great, but optional of course, to add an image to Figure 1 that shows some of the described morphological characteristics.

Are sufficient details of methods and materials provided to allow replication by others?

Yes

Is the rationale for creating the dataset(s) clearly described?

Yes

Are the datasets clearly presented in a useable and accessible format?

Yes

Are the protocols appropriate and is the work technically sound?

Yes

Reviewer Expertise:

genomics

I confirm that I have read this submission and believe that I have an appropriate level of expertise to confirm that it is of an acceptable scientific standard, however I have significant reservations, as outlined above.

# Response

**Published:** 2025-09-20

**Authors:** Tree of Life Team Sanger

**Affiliations:** Wellcome Trust Sanger Institute, UK

Thank you for reviewing this data note. We have made changes to version 2 of the article, and respond to your main points below:

**Reviewer comment: “A karyotype is likely not known (?) and the HiC signal shows variation inside the chromosome-level scaffolds. E.g. scaffold 2 and 5 have a block at the bottom that equal signal to the other regions in same same and other scaffolds. Scaffold 1 also has a smaller part at the bottom that appears different. I therefore wonder if one can check if the 6 chromosomes have telomers and if the potentially problematic regions lack telomers.”**

*Response: We curated the assembly in PretextView with a telomere-repeat track enabled to help verify chromosome boundaries. The regions highlighted by the reviewer do not show off-diagonal contact patterns indicative of mis-joins; the diagonals remain continuous through these areas.* *We have replaced the static image of the Hi-C chromosome map with a labelled PretextView map. The HiGlass image remains hyperlinked in the caption.*

**Reviewer comment: “What is meant by 'dubious records' in the introduction? Species misidentification?”**

*Response: We have clarified in the text that we are referring to potentially misidentified specimens. *   The Tree of Life team

# Reviewer report: Reviewer response for version 1

**DOI:** 10.21956/wellcomeopenres.26221.r120912

**Published:** 2025-03-31

**Authors:** Nerivânia Nunes Godeiro

**Affiliations:** 1 Shanghai Natural History Museum, Shanghai, Shanghai, China

The authors presented the genome of *Ochesella flavescens*, a Collembola species widespread in Europe. 

 In the background section, the authors said that the species belongs to Entomobryidae family, but it belongs to Orchesellidae, as described in the species taxonomy section.

 I have more concerns about the methodology. The first is related to the BUSCO reference set used to assess the completeness of the genome. There is a specific reference set  "collembola_odb10" (n=4073) available, and the authors used a general "arthropoda_odb10" (n=1013).

 I also didn't understand how the authors were able to perform all experiments using only one specimen, at least this is what they said in the sample acquisition topic. Sometimes one whole specimen is not enough to produce a good result, imagine a bit of tissue. It would be interesting to see the protocol followed for this specific case, not the general protocol cited as the reference. Collembola are tiny animals, please give more details of how much tissue was used for each step. 

 The mitogenome sequence submitted to NCBI was not annotated. Did you check if it's complete? 

 Except for these details, the article is well written and makes an important contribution.

Are sufficient details of methods and materials provided to allow replication by others?

Partly

Is the rationale for creating the dataset(s) clearly described?

Partly

Are the datasets clearly presented in a useable and accessible format?

Yes

Are the protocols appropriate and is the work technically sound?

Yes

Reviewer Expertise:

Collembola phylogenomics and taxonomy

I confirm that I have read this submission and believe that I have an appropriate level of expertise to confirm that it is of an acceptable scientific standard, however I have significant reservations, as outlined above.

# Response

**Published:** 2025-09-20

**Authors:** Tree of Life Team Sanger

**Affiliations:** Wellcome Trust Sanger Institute, UK

Thank you for reviewing this data note. We have made changes to version 2 of the article, and respond to your main points below:  

**Reviewer comment 1:** In the background section, the authors said that the species belongs to Entomobryidae family, but it belongs to Orchesellidae, as described in the species taxonomy section.

*Response: We have corrected this and added an explanation in the background about the change of taxonomic assignment for this species. *  

**Reviewer comment 2: **I have more concerns about the methodology. The first is related to the BUSCO reference set used to assess the completeness of the genome. There is a specific reference set  "collembola_odb10" (n=4073) available, and the authors used a general "arthropoda_odb10" (n=1013).

*Response: There is no officially released BUSCO lineage for Collembola in either ODB10 or ODB12; the BUSCO catalogue provides higher-level sets (e.g. arthropoda_odb10) but not a Collembola-specific dataset. Accordingly, we used the closest available official lineage on our system, which is arthropoda_odb10 for ODB10, to ensure reproducibility.*

**Reviewer comment 3:** I also didn't understand how the authors were able to perform all experiments using only one specimen, at least this is what they said in the sample acquisition topic. Sometimes one whole specimen is not enough to produce a good result, imagine a bit of tissue. It would be interesting to see the protocol followed for this specific case, not the general protocol cited as the reference. Collembola are tiny animals, please give more details of how much tissue was used for each step. 

*Response: It is difficult to obtain enough material for both PacBio and DNA sequencing from a tiny arthropod, but it is preferable to use Hi-C data from the same organism to scaffold the assembly. The Methods section indicates that we used the Ultra-low input library preparation technique and Revio sequencing to achieve good genome coverage.*

**Reviewer comment 4:** The mitogenome sequence submitted to NCBI was not annotated. Did you check if it's complete? 

*Response: Yes, while we do not provide full annotation, MitoHiFi uses MitoFinder to ensure that the sequence is complete.*

# Reviewer report: Reviewer response for version 1

**DOI:** 10.21956/wellcomeopenres.26221.r120921

**Published:** 2025-03-31

**Authors:** Daoyuan Yu, Zhihong Zhan

**Affiliations:** 1 Nanjing Agricultural University, Nanjing, China

This study provides foundational data for the field of springtail genomics.

**Below are my review comments:**

1. A detailed analysis of repeat sequence proportions and types should be provided.
2. BUSCO assessment shows 7.6% duplicated genes, exceeding the EBP standard of less than 5%; however, no explanation is provided.
3. No detailed discussion of how genome heterozygosity (0.3%) affected assembly.
4. A higher-resolution version of the Hi-C contact map in Figure 5 is needed.
5. Table 3 should include information on gene density for each chromosome.

Are sufficient details of methods and materials provided to allow replication by others?

Yes

Is the rationale for creating the dataset(s) clearly described?

Yes

Are the datasets clearly presented in a useable and accessible format?

Yes

Are the protocols appropriate and is the work technically sound?

Yes

Reviewer Expertise:

Collembola systematics, ecology and genomics

We confirm that we have read this submission and believe that we have an appropriate level of expertise to confirm that it is of an acceptable scientific standard, however we have significant reservations, as outlined above.
